# Primary adrenal insufficiency is associated with impaired natural killer cell function: a potential link to increased mortality

**DOI:** 10.1530/EJE-16-0969

**Published:** 2017-01-23

**Authors:** Irina Bancos, Jon Hazeldine, Vasileios Chortis, Peter Hampson, Angela E Taylor, Janet M Lord, Wiebke Arlt

**Affiliations:** 1 Institute of Metabolism and Systems ResearchUniversity of Birmingham, Birmingham, UK; 2 Division of EndocrinologyDiabetes, Metabolism, and Nutrition, Department of Internal Medicine, Mayo Clinic, Rochester, Minnesota, USA; 3 Institute of Inflammation and Ageing; 4 Medical Research Council-Arthritis Research UK (MRC-ARUK) Centre for Musculoskeletal Ageing ResearchUniversity of Birmingham, Birmingham, UK; 5 Centre for EndocrinologyDiabetes and Metabolism, Birmingham Health Partners, Birmingham, UK

## Abstract

**Objective:**

Mortality in patients with primary adrenal insufficiency (PAI) is significantly increased, with respiratory infections as a major cause of death. Moreover, patients with PAI report an increased rate of non-fatal infections. Neutrophils and natural killer (NK) cells are innate immune cells that provide frontline protection against invading pathogens. Thus, we compared the function and phenotype of NK cells and neutrophils isolated from PAI patients and healthy controls to ascertain whether altered innate immune responses could be a contributory factor for the increased susceptibility of PAI patients to infection.

**Design and methods:**

We undertook a cross-sectional study of 42 patients with PAI due to autoimmune adrenalitis (*n = *37) or bilateral adrenalectomy (*n = *5) and 58 sex- and age-matched controls. A comprehensive screen of innate immune function, consisting of measurements of neutrophil phagocytosis, reactive oxygen species production, NK cell cytotoxicity (NKCC) and NK cell surface receptor expression, was performed on all subjects.

**Results:**

Neutrophil function did not differ between PAI and controls. However, NKCC was significantly reduced in PAI (12.0 ± 1.5% vs 21.1 ± 2.6%, *P* < 0.0001). Phenotypically, the percentage of NK cells expressing the activating receptors NKG2D and NKp46 was significantly lower in PAI, as was the surface density of NKG2D (all *P* < 0.0001). Intracellular granzyme B expression was significantly increased in NK cells from PAI patients (*P* < 0.01).

**Conclusions:**

Adrenal insufficiency is associated with significantly decreased NKCC, thereby potentially compromising early recognition and elimination of virally infected cells. This potential impairment in anti-viral immune defense may contribute to the increased rate of respiratory infections and ultimately mortality in PAI.

## Introduction

Primary adrenal insufficiency (PAI), or Addison’s disease, is characterized by the insufficient production of mineralocorticoids, glucocorticoids and androgen precursors by the adrenal cortex. Daily corticosteroid replacement therapy is the standard treatment for adrenal failure, and since its introduction following the Nobel Prize-winning achievements of Kendall, Hench and Reichstein (1), the life expectancy of PAI patients has increased considerably (2, 3). However, despite ready availability of corticosteroid replacement, Addison’s patients not only suffer from a suboptimal quality of life (4, 5) but also from an increased risk of premature death, with infections reported as a major cause (6, 7). A population-based, retrospective, observational study in patients with Addison’s disease (6) found a two-fold increased risk ratio for death and a five times higher mortality rate from infections than in the general population, with pneumonia being the major cause of infection-related death. In line with these observations, Erichsen *et al*. (7) subsequently reported an increased standard mortality rate (SMR) for PAI patients, with infections being the cause of death in 10% of these patients, compared to 6% in the general population. Infectious diseases have also been documented as a main cause of death in patients with secondary adrenal insufficiency due to hypopituitarism, when compared to hypopituitarism patients with normal hypothalamic–pituitary–adrenal axis function (SMR 8.88) (8).

Infection is an acute process that triggers a cytokine-mediated inflammatory stress response including an increased requirement for glucocorticoid activity; therefore, in adrenal insufficiency, a superimposed adrenal crisis can contribute to the observed increase in mortality arising from infections. Indeed, most of adrenal crises are precipitated by an infection as the trigger, and in the majority of cases, underlying viral infections have been documented (3, 9). Patients with adrenal insufficiency have also been reported to suffer from an increased rate of non-fatal infections, with a 1.5-fold increased risk of use of anti-microbial agents and a 4.0- to 5.0-fold increased risk of hospital admission for infection (10), with rates of pneumonia reported to be more than nine times higher in adrenal insufficiency patients. However, despite these well-documented reports about increased incidence and severity of infection among PAI patients, it is currently unknown as to why these individuals succumb more readily to infections when compared to the general population.

Neutrophils and natural killer (NK) cells are critical effector cells of the innate immune system and serve as the first line of defense against viral, fungal and bacterial infections. We hypothesized that an impairment in the function of either or both of these major cell types underlies the increased susceptibility of PAI patients to infection. Neutrophils provide immediate frontline anti-microbial protection by phagocytosis of microbes and the generation of reactive oxygen species (ROS), whereas NK cell-mediated elimination of virus-infected cells is achieved primarily through contact-dependent cytotoxicity. Thus, the objective of this cross-sectional study was to examine the innate immune response in adrenal insufficiency. To this end, we analyzed both the function and surface phenotype of neutrophils and NK cells in a cohort of patients with PAI receiving chronic corticosteroid replacement in comparison to sex- and age-matched healthy controls.

## Subjects and methods

### Subjects

We carried out a cross-sectional study with prospective enrolment of patients with an established diagnosis of PAI and sex- and age-matched healthy controls, with total recruitment taking place over a period of 18 months. Exclusion criteria were acute illness, immunological disease, significant comorbidities likely to influence immune function and intake of drugs that are known to affect steroid synthesis, metabolism or immunity. Subjects provided written informed consent prior to the enrolment and after the approval of the study protocol by the local research ethics committee. Blood samples were taken in the morning (09:00–11:30 h), which in the PAI patients equated to 2–5 h after intake of their regular morning glucocorticoid replacement dose. In addition to taking a detailed history during the study visit, medical records were reviewed to obtain clinical information.

### Steroid hormone analysis

Steroids were extracted from 200 µL of serum by liquid/liquid extraction using MTBE (tert-methyl butyl ether) as described previously (11). DHEA sulfate (DHEAS) was extracted from 20 µL of serum after protein precipitation as described by Chadwick *et al*. (12).

A Waters Xevo Mass Spectrometer with an electrospray ionization source (in positive ionization mode) and an attached Acquity liquid chromatography system was used to identify and quantify the steroids. Steroids were eluted using a HSS T3, 1.8 µm, 1.2 × 50 mm column using an optimized methanol/water 0.1% formic acid gradient system. After initial analysis, samples were evaporated and derivatized to form oxime derivatives to improve the sensitivity to DHEA.

For accurate quantitation using liquid chromatography–tandem mass spectrometry, two mass transitions for each steroid analyte and its isotopically labeled internal standard were defined, followed by quantification facilitated by referring to a calibration series spanning the expected concentration range for unconjugated steroids, 0.25–500 ng/mL, and for DHEA sulfate (DHEAS), 0.25–10 µg/mL.

### Isolation of immune cells

Peripheral blood mononuclear cells (PBMCs) were isolated from whole blood by Ficoll density gradient centrifugation. NK cells were isolated from PBMCs by negative selection using magnetic assisted cell sorting (MACS) technology according to manufacturer’s instructions (Miltenyi Biotec, Gladbach, Germany). NK cells routinely constituted ≥96% of the isolated cell population.

### Assessment of neutrophil function and surface phenotype

#### Measurement of neutrophil phagocytosis and reactive oxygen species (ROS) production

Following manufacturer’s instructions of the commercially available PhagoTEST and PhagoBURST kits (BD Biosciences), neutrophil phagocytosis of opsonized *Escherichia coli* (*E. coli*) and ROS production in response to *E. coli* stimulation respectively, were measured in 100 µL aliquots of heparinized whole blood. For both assays, 10 000 neutrophils were analyzed on an Accuri C6 flow cytometer and data were evaluated using CFlow software (BD Biosciences). Phagocytosis was recorded as the percentage of neutrophils that had phagocytosed bacteria and the number of bacteria engulfed per cell (mean fluorescent intensity (MFI)). Using both measures, the phagocytic index (PI) of neutrophils was calculated as (% of neutrophils that had ingested bacteria/100) × MFI.

#### CD16 surface expression

100 µL aliquots of heparinized whole blood were immunostained for 20 min on ice with 4 µg/mL CD16-APC-conjugated antibody (Clone 3G8; BD Biosciences) or its concentration-matched isotype control. After incubation, red blood cells were lysed (BD FACS Lyse solution, BD Biosciences), samples were washed in phosphate-buffered saline (PBS) and CD16 expression on 10 000 neutrophils was analyzed on an Accuri C6 flow cytometer.

### Assessment of natural killer (NK) cell function and surface phenotype

#### Phenotypic analysis of PBMC samples

PBMCs (2 × 10^5^) were immunostained on ice for 20 min with combinations of the following fluorochrome-conjugated mouse monoclonal antibodies or their concentration-matched isotype controls: 1 µg/mL CD3-Pacific Blue (Clone UCHT1; BD Biosciences), 1 µg/mL CD3-PeCy7 (Clone UCHT1; eBioscience, Hatfield, UK), 1 µg/mL CD56-PE (Clone AF12-7H3; Miltenyi Biotec), 5 µg/mL NKp46-Pacific Blue (Clone 9E2; BioLegend, Cambridge, UK) or 10 µg/mL NKG2D-PeCy7 (Clone 1D11; BioLegend). After incubation, samples were washed in PBS prior to flow cytometric analysis on a CyAN_ADP_ cytometer (Dako, Cambridgeshire, UK) where receptor expression was studied on 5000 CD3^−^ CD56^DIM^ NK cells.

#### Measurement of NK cell activating receptor expression on isolated NK cells

Resting NK cells (2 × 10^5^) were immunostained for 20 min on ice with 5 µg/mL of purified mouse anti-human NKp30 antibody (Clone P30-15; BioLegend) or its concentration-matched isotype control (Clone MOPC-21; BioLegend). After a single wash in PBS, samples were resuspended in PBS/1% bovine serum albumin (BSA) containing 20% (vol/vol) goat serum (Sigma-Aldrich) and incubated for 20 min on ice, after which cells were washed in PBS and pellets were resuspended in PBS/1%BSA containing 10 µg/mL of FITC goat anti-mouse IgG (Clone Poly4053; BioLegend). After a 20-min incubation on ice, cells were washed in PBS and analyzed on a CyAN_ADP_ cytometer, where 10 000 NK cells were assessed.

#### Intracellular staining for the assessment of perforin and granzyme B expression

Resting NK cells (2 × 10^5^) were fixed for 30 min at room temperature (RT) in fixation medium (Life Technologies). After incubation, samples were washed in PBS and resuspended in permeabilization medium (Life Technologies) that contained 10 µg/mL of a FITC-conjugated monoclonal antibody against human perforin (Clone δG9; BioLegend), 16 µg/mL of a FITC-conjugated monoclonal antibody against human granzyme B (Clone GB11; BioLegend) or their concentration-matched isotype controls. Samples were incubated for 30 min at RT and then washed in PBS prior to flow cytometric analysis, where on a CyAN_ADP_ cytometer (Dako), 10 000 NK cells were studied.

#### NK cell cytotoxicity (NKCC) assay

NKCC was assessed using a modified version of the protocol described by Godoy-Ramirez *et al*. (13). Briefly, K562 target cells were cultured either alone or with NK cells at an effector:target (E:T) cell ratio of 10:1 for 4 h at 37°C in a humidified 5% CO_2_ atmosphere. After incubation, cells were pelleted and resuspended in PBS/1%BSA containing 0.3 µg anti-CD56 PE (Miltenyi Biotec). Following a 10-min incubation on ice, samples were washed in PBS and stained for 5 min with 125 nM of the dead cell stain sytox blue (Life Technologies) prior to flow cytometric analysis.

To measure NKCC, the number of lysed K562 target cells (defined as sytox blue positive) in a total population of 2000 was recorded. From here, the percentage of specific cell lysis was calculated as: (TL-SL/2000) × 100, where TL is the number of lysed target cells in NK-K562 co-culture samples and SL is the number of K562 cells that underwent lysis when cultured with media alone. To measure the target cell–NK cell conjugate formation rate, conjugates were defined as K562 cells exhibiting positive PE fluorescence and the number in a population of 2000 K562 cells was recorded.

### Statistical analysis

Statistical analyses were performed using GraphPad Prism software (GraphPad Software Ltd) and JMP software, version 10 (SAS Institute Inc., Cary, NC, USA). Continuous data are summarized as mean and standard deviation (s.d.) or median and ranges, depending on whether or not they were normally distributed, whereas categorical data are summarized as number (%). Associations between AI and subject characteristics were assessed using the Kruskal–Wallis test for continuous variables and the chi-square test for categorical variables. Data distribution was examined using the Kolmogorov–Smirnov test. For data that followed a normal distribution, unpaired Student *t* tests were performed, whereas for non-normally distributed data, a Mann–Whitney *U* test was used. In dot plots, horizontal lines represent the median value. Statistical significance was accepted at *P* ≤ 0.05.

## Results

### Clinical phenotype and demographics in PAI patients and controls

We recruited 43 PAI patients (35 females, 8 males) with a median age of 49 (range: 18–79) years (men: 46.5 (18–64) years; women: 49 (19–79) years) (for details see Table 1). Median BMI was 23.5 (19–30) kg/m^2^ in the male patients and 25 (19–41) kg/m^2^ in the women with PAI. Steroid replacement therapy consisted of hydrocortisone (median daily dose 25 (range: 10–40) mg) and fludrocortisone (daily dose 100 (50–300) µg). Most patients were taking hydrocortisone in two separate daily doses (27/43; 63%), with the remainder taking once-daily (3/43; 7%) or thrice-daily doses (13/43; 30%). Eleven of the 35 women with PAI were on long-standing replacement with the androgen precursor DHEA (daily dose: 25 (25–50) mg), and intake was confirmed by circulating serum DHEAS (women without DHEA replacement 0.2 (0.1–0.2) µmol/L vs 6.7 (3.2–8.3) µmol/L in women on DHEA replacement; *P* < 0.0001).

**Table 1:** Clinical characteristics of the 43 patients with primary adrenal insufficiency included in this study.

**Patient number**	**Sex** (F/M)	**Age** (years)	Body mass index (kg/m^2^)	**Hydro-cortisone daily dose** (mg)	**Number of daily hydro-cortisone doses**	**Fludro-cortisone once daily dose** (g)	**DHEA once daily dose** (mg)	**Duration of disease** (years)	**Cause of primary adrenal insufficiency**
1	F	19	28.3	20	3	150	–	3	Polyglandular syndrome type 2
2	F	20	19.0	20	2	150	–	4	Polyglandular syndrome type 2
3	F	28	32.3	20	2	200	25	9	Polyglandular syndrome type 2
4	F	30	34.5	25	2	100	–	11	Isolated Addison’s disease
5	F	35	21.2	25	2	100	–	12	Isolated Addison’s disease
6	F	35	23.0	25	2	200	25	11	Isolated Addison’s disease
7	F	36	26.5	30	2	250	–	23	Isolated Addison’s disease
8	F	40	21.9	20	2	150	–	17	Polyglandular syndrome type 2
9	F	40	29.0	25	2	150	37.5	5	Polyglandular syndrome type 2
10	F	40	37.2	20	2	300	–	27	Isolated Addison’s disease
11	F	42	22.1	25	2	125	–	11	Polyglandular syndrome type 2
12	F	42	41.0	20	1		–	13	Isolated Addison’s disease
13	F	44	35.0	40	3	100	–	1	Isolated Addison’s disease
14	F	45	23.3	15	2	50	50	14	Polyglandular syndrome type 2
15	F	46	22.9	30	2	100	–	3	Polyglandular syndrome type 2
16	F	48	23.0	25	3	100	–	20	Polyglandular syndrome type 2
17	F	49	20.3	25	2	150	–	26	Polyglandular syndrome type 2
18	F	53	23.0	20	3	100	25	12	Isolated Addison’s disease
19	F	54	27.4	30	2	50	25	17	Polyglandular syndrome type 2
20	F	55	23.0	20	2	100	25	13	Polyglandular syndrome type 2
21	F	56	21.0	30	2	200	–	11	Isolated Addison’s disease
22	F	56	29.4	22.5	3	250	–	30	Polyglandular syndrome type 2
23	F	60	21.2	25	3	100	37.5	12	Polyglandular syndrome type 2
24	F	63	24.7	10	1	100	–	28	Polyglandular syndrome type 2
25	F	63	21.0	15	2	50	–	18	Polyglandular syndrome type 2
26	F	67	29.3	20	2	100	25	30	Polyglandular syndrome type 2
27	F	69	24.4	30	2	100	–	49	Isolated Addison’s disease
28	F	71	25.3	20	1	100	–	31	Polyglandular syndrome type 2
29	F	73	25.0	20	3		–	3	Polyglandular syndrome type 2
30	F	77	26.6	20	3	100	–	24	Polyglandular syndrome type 2
31	F	79	28.0	30	3	50	–	38	Isolated Addison’s disease
32	F	43	18.8	30	2	250	25	1	Bilateral adrenalectomy
33	F	49	25.0	25	2	150	–	7	Bilateral adrenalectomy
34	F	64	25.0	20	3	100	–	12	Bilateral adrenalectomy
35	F	55	31.7	20	2	–	50	27	Adrenalectomy for Cushing
36	M	18	22.1	25	3	150	–	1	Isolated Addison’s disease
37	M	19	19.4	25	2	100	–	1	Polyglandular syndrome type 2
38	M	38	23.0	40	3	150	–	14	Isolated Addison’s disease
39	M	59	20.6	30	3	150	–	8	Polyglandular syndrome type 2
40	M	63	28.0	30	2	100	–	29	Isolated Addison’s disease
41	M	64	30.0	30	2	150	–	29	Isolated Addison’s disease
42	M	30	24.0	30	2	300	–	6	Bilateral adrenalectomy
43	M	55	28.2	25	2	100	–	4	Bilateral adrenalectomy

Underlying cause of disease in the PAI patients was autoimmune adrenalitis (*n = *37; 15 patients with isolated autoimmune Addison’s disease, 22 with autoimmune polyglandular syndrome type 2, APS2), permanent glucocorticoid deficiency after adrenalectomy for Cushing syndrome in one patient and bilateral adrenalectomy in the five remaining patients. Duration of PAI since diagnosis was established for the first time was 13 (0.5–49) years. Comorbidities in the 22 patients with APS2 included autoimmune thyroid disease (*n = *18), pernicious anemia (*n = *3), premature ovarian failure (*n = *4), type 1 diabetes (*n = *2), vitiligo (*n = *2) and asthma (*n = *2). All APS2 patients had a family history of autoimmunity comprising thyroid disease (*n = *18), ulcerative colitis/Crohn’s disease (*n = *5), type 1 diabetes mellitus (*n = *4), vitiligo (*n = *3), PAI (*n = *2) and pernicious anemia (*n = *1).

We recruited 27 healthy controls, 12 men with a median age of 41 (31–61) years and 15 women aged 42 (34–50) years. In addition, we recruited 31 patients from the Birmingham 1000 Elders cohort http://www.birmingham.ac.uk/research/activity/mds/centres/healthy-ageing/elders.aspx as healthy elderly controls (age: 73 (62–91) years).

### Neutrophil function in PAI is normal

ROS generation and phagocytosis are two key microbicidal mechanisms of neutrophils. When challenged with immunoglobulin (Ig) and complement-coated *E. coli*, no difference was found in ROS generation between neutrophils from PAI patients and age- and sex-matched healthy controls (Table 2). However, a trend (*P *= 0.07) toward a reduced phagocytic index for neutrophils from PAI patients was observed (Table 2). A measure of phagocytic activity, phagocytic index, takes into account both the percentage of neutrophils that have ingested bacteria as well as their individual phagocytic activity (number of bacteria per cell). Analysis of the phagocytic activity revealed a trend (*P *= 0.08) toward reduced phagocytic activity of neutrophils from PAI patients, with no difference found in respect to the percentage of neutrophils capable of ingesting *E.coli* (Table 2).

**Table 2:** Neutrophil phagocytosis, reactive oxygen species (ROS) generation and surface CD16 expression in 42 PAI patients and 27 healthy, sex- and age-matched controls. For the assessment of CD16 expression, 41 PAI patients and 27 healthy controls were studied. Values are presented as mean ± standard deviation.

	**PAI patients**	**Healthy controls**	***P***
Phagocytic index	257 252 ± 87 162	299 220 ± 98 779	0.07
% Phagocytosing neutrophils	98.0 ± 1.5	98.2 ± 1.2	0.68
Phagocytic activity (MFI)	262 469 ± 88 707	304 941 ± 100 876	0.08
ROS generation (MFI)	57 023 ± 26 463	49 915 ± 19 302	0.23
% CD16^+^ neutrophils	97.46 ± 1.82	96.63 ± 3.04	0.33
CD16 surface density	58 775 ± 27 559	68 380 ± 31 401	0.20

MFI, median fluorescence intensity. Phagocytic index calculated as (% phagocytosis/100) × phagocytic activity.

Surface expression of the F_C_ receptor CD16 is critical for neutrophils to phagocytose Ig-coated pathogens. Thus, having observed a trend for impaired phagocytosis by neutrophils from PAI patients, we compared surface expression of CD16 on neutrophils isolated from patients and healthy sex- and age-matched controls, which, however, revealed no difference between PAI patients and controls (Table 2).

### Natural killer cell cytotoxicity is decreased in PAI

**Figure 1:**
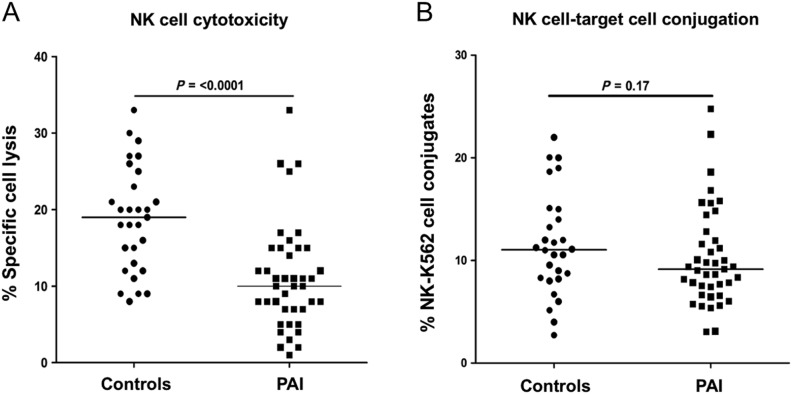
Natural killer cell cytotoxicity and conjugate formation. Panel A, cytotoxicity of resting NK cells isolated from PAI patients (*n = *41) and age- and sex-matched healthy controls (*n = *29) toward the erythroleukemic K562 cell line at an effector:target cell ratio of 10:1. Horizontal line depicts the median value. Panel B, percentage of NK cells bound to K562 tumor cells as a measure of conjugate formation between effector (NK) and target (K562) cells. Conjugate formation was assessed following a 4-h co-culture at an effector:target cell ratio of 10:1. Data were obtained from 41 PAI patients and 28 age- and sex-matched healthy controls. Horizontal line depicts the median value.

NK cells rapidly eliminate virally infected cells via contact-dependent cytotoxicity, which begins with conjugate formation between NK cells and the infected target cell. Through the use of two-color flow cytometry, we performed a simultaneous assessment of conjugate formation and natural killer cell cytotoxicity (NKCC). NK cells from PAI patients exhibited significantly reduced cytotoxicity toward transformed cells when compared to NK cells from healthy controls (PAI: 12.0 ± 1.5% vs 21.1 ± 2.6% in controls, *P* < 0.0001) (Fig. 1A), whereas both groups exhibited similar ability for NK cell–target cell conjugate formation (Fig. 1B).

### Natural killer granzyme B expression is increased in PAI

**Figure 2:**
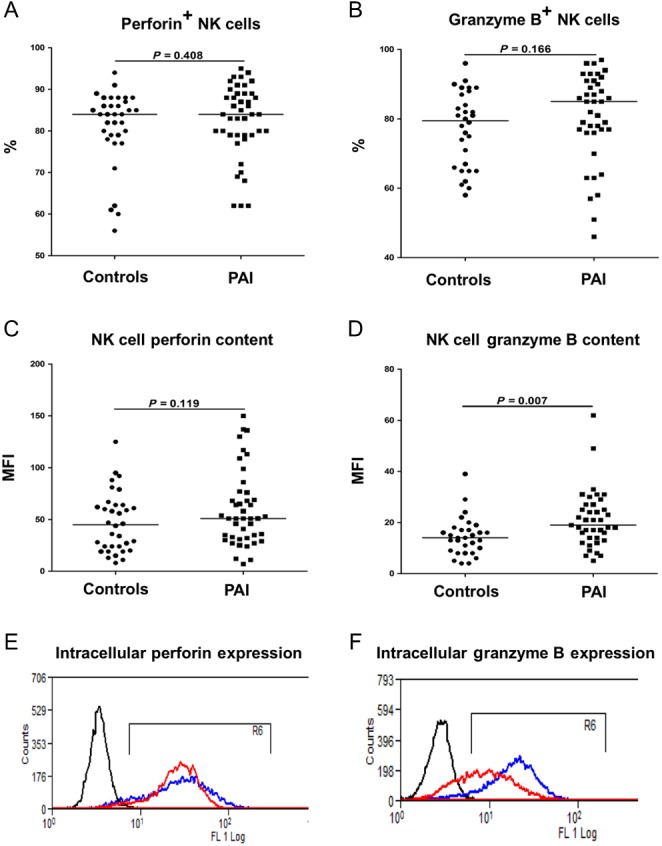
Intracellular perforin and granzyme B expression in resting NK cells. Panels A and C, NK cells isolated from PAI patients (*n = *42) and age- and sex-matched healthy controls (*n = *34) were analyzed for intracellular perforin expression. Data are presented as percentage positive NK cells (Panel A) and staining intensity (Panel C). Horizontal line depicts the median value. Panels B and D, NK cells isolated from PAI patients (*n = *39) and age- and sex-matched healthy controls (*n = *30) were analyzed for intracellular granzyme B expression. Data are presented as percentage positive NK cells (Panel B) and staining intensity (Panel D). Horizontal line depicts the median value. MFI, mean fluorescence intensity. Panels E and F, representative flow cytometry plots depicting intracellular perforin (Panel E) and granzyme B (Panel F) expression in NK cells isolated from a single PAI patient (blue line) and healthy control (red line). The black line represents the isotype control.

Granule exocytosis is the predominant mechanism by which NK cells mediate target cell lysis. Two molecules central to this form of NK cell defense are the pore-forming protein perforin and the serine protease granzyme B. We found no difference in the percentage of perforin or granzyme B-positive NK cells between PAI patients and their age- and sex-matched controls (Fig. 2A and B). Similarly, no differences were observed in the staining intensity (median fluorescence intensity) of perforin between PAI and controls (59.9 ± 36.4 vs 47.0 ± 28.7; *P* = 0.12) (Fig. 2C and E). However, the staining intensity of granzyme B was found to be significantly higher in NK cells from PAI patients than that in controls (21.1 ± 11.1 vs 14.5 ± 7.5; *P *= 0.007) (Fig. 2D and F).

### Natural killer cell-activating receptor expression is decreased in PAI

The induction of NKCC is governed in part by signals transmitted through surface-expressed NK cell-activating receptors, which recognize ligands present on the surface of target cells. To determine whether altered NK cell-activating receptor expression could explain the impaired NKCC in PAI patients, we measured the surface expression of the NK cell-activating receptor NKG2D and the natural cytotoxicity receptors NKp30 and NKp46. Compared to healthy controls, the percentage of NK cells expressing NKG2D and NKp46 was significantly lower in PAI patients as was the surface density of NKG2D (all *P* < 0.0001). In addition, a trend (*P* = 0.06) for a reduced frequency of NKp30-positive NK cells was observed (Table 3).

**Table 3:** Expression of activatory surface receptors on the surface of natural killer (NK) cells. For NKG2D, NK cells isolated from 42 PAI patients and 30 healthy controls were studied. For NKp30, *n* = 40 for PAI patients and *n *= 30 for healthy controls. For NKp46, we studied NK cells from 42 PAI patients and 32 healthy controls. Values are presented as mean ± standard deviation. Significant differences are indicated in bold font.

	**Percentage positive NK cells**			**Surface density** (MFI)		
	Healthy controls	PAI patients	*P*	Healthy controls	PAI patients	*P*
NKG2D	92.6 ± 4.3	78.0 ± 11.9	**<0.0001**	34.8 ± 7.9	20.4 ± 5.8	**<0.0001**
NKp30	65.6 ± 20.5	56.4 ± 19.6	0.06	14.5 ± 8.8	13.6 ± 8.0	0.34
NKp46	46.8 ± 212	27.9 ± 14.0	**<0.0001**	7.6 ± 4.0	6.7 ± 2.9	0.32

MFI, mean fluorescence intensity.

## Discussion

Although infections are a common occurrence among patients with PAI (3, 9, 10) and a major cause of the excess mortality that is observed among this patient group (6, 7), it is currently unknown as to why these individuals report more frequent and severe infectious episodes when compared to the general population. Here, we have carried out a comparative assessment of the function and surface phenotype of NK cells and neutrophils isolated from PAI patients and age- and sex-matched healthy controls in an effort to ascertain whether defects in innate immunity could explain the increased susceptibility of PAI patients to infection.

 As the first immune cell to arrive at a site of infection, neutrophils provide immediate frontline protection against rapidly dividing bacteria, fungi and yeast. Based on the results of a recent study that reported an increased incidence of fungal infection in PAI patients (10), we hypothesized that neutrophils from these patients would exhibit impaired microbicidal activity when compared to healthy controls. Although no difference was found in ROS generation between the two groups, we observed a trend (*P* = 0.07) toward reduced phagocytosis of opsonized *E.coli* by neutrophils from PAI patients, an impairment that could negatively influence their ability to eliminate invading pathogens, though clearly this would appear not to be a major contributor to the increased incidence of infection among this group.

In contrast, we have shown for the first time that NK cells isolated from patients with PAI exhibit significantly impaired cytotoxicity at the single cell level. As NK cells are involved in the early recognition and elimination of virus-infected cells, this defect in NKCC may be one factor underlying the increased incidence and severity of viral infections reported by PAI patients (10). Interestingly, a recent report has described reduced innate anti-viral responses in PBMCs in PAI, specifically reduced CXCL9 and CXCL10 production in response to stimulation with interferon (14). Taking those findings together with our data, we are now looking at significant evidence that there is a major defect in the innate immune response in PAI, potentially increasing susceptibility to viral infections.

**Figure 3:**
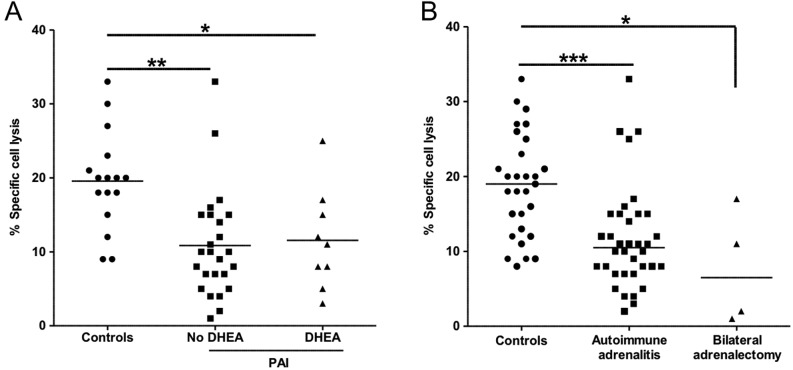
Natural killer cell cytotoxicity (NKCC), DHEA replacement therapy and cause of primary adrenal insufficiency (PAI). Panel A shows NKCC (median and individual data points) as assessed in healthy controls (*n = *29) and patients with PAI (*n = *41), separated into patients with (*n = *11) and without (*n = *30) chronic DHEA replacement therapy. Panel B shows NKCC (median and individual data points) in healthy controls (*n = *29), patients with PAI due to autoimmune adrenalitis (*n = *36) and patients with PAI after bilateral adrenalectomy (*n = *4). Statistical comparisons were made with a non-parametric Kruskal–Wallis test with Dunn’s multiple comparison test. *P < 0.05; **P < 0.01; ***P < 0.001.

The adrenal androgen precursor dehydro­epiandrosterone (DHEA) has been reported to increase NK cell numbers (15, 16), albeit mainly in rodent-based studies, which complicates matters as rodent adrenals do not produce DHEA in substantial amounts. One human-based study has examined the immunological effects of twelve-week dehydroepiandrosterone replacement therapy in patients with PAI (17) and found that DHEA treatment reduced the frequency of NK and NKT cells. However, the study did not comment on baseline differences in NK cell function or frequency between patients and healthy controls. In our studies, we found no difference in circulating NK cell numbers between PAI patients and healthy controls, but found significantly decreased NKCC, which was equally reduced in patients with and without long-standing DHEA replacement therapy (Fig. 3A), excluding DHEA deficiency as the causative factor explaining the observed decrease in NKCC.

Glucocorticoids have been shown to potently suppress NKCC in a series of *in vitro* studies (18, 19, 20). Current glucocorticoid replacement options are not able to mimic the physiologic circadian cortisol secretion but result in non-physiological peak and trough cortisol concentrations equating to transient over- and under-replacement, which could impact on NK cell function through altered CLOCK gene regulation of immune function. NK cells of mice with deletion of the clock gene period circadian clock 1 (Per1) display significantly altered diurnal rhythms of cytokine release and expression of the cytolytic factors perforin and granzyme B (21). This resonates with our finding of increased granzyme B expression in the NK cells of our PAI patients. Moreover, in a recent paper, it has been shown that oral hydrocortisone in doses of 20 mg acutely upregulate PER1 in human peripheral blood mononuclear cells and that glucocorticoid pulses can be used to entrain an aberrant diurnal rhythm (22).

The induction of NKCC is governed in part by signals transmitted through surface-expressed germline-encoded activatory receptors (23). Upon ligand recognition, NK cell-activating receptors, which include the natural cytotoxicity receptors NKp30 and NKp46 and the C-type lectin family member NKG2D, initiate diverse signaling pathways, which provided they overcome signals from inhibitory receptors lead to NK cell activation (24). For the cytotoxicity assays performed in this study, the MHC class I-deficient erythroleukemic K562 cell line was used as the target cell. Signaling through the NK cell-activating receptor NKG2D has been shown to be primarily responsible for NK-mediated lysis of K562 cells (25). Compared to healthy controls, we found that both the frequency of NKG2D-positive NK cells and its surface density were significantly lower in PAI patients, highlighting a potential mechanistic explanation for the reduced cytotoxicity of NK cells from PAI patients against K562 cells. In addition to NKG2D, we found a significantly reduced frequency of circulating NKp46-positive NK cells among PAI patients and a trend (*P* = 0.06) for a reduced percentage of NKp30-positive NK cells. Although changes in NKp46 and NKp30 expression are unlikely to be responsible for the impaired cytotoxicity against K562 cells we have described in this report (25), they may contribute to the increased susceptibility of PAI patients to infection. For example, NKp46 has been shown to directly bind the hemagglutinin of influenza virus (26) and to detect human monocytes infected with mycobacterium tuberculosis (TB) (27). Thus, a reduced frequency of NK cells expressing NKp46 may explain to some degree the increased incidence of viral infections among PAI patients (10) and may also hamper the immune response against TB, which represents a major cause of PAI disease in the developing world. In respect of NKp30, this receptor has been shown to play an essential role in NK cell-dependent maturation of dendritic cells (DCs) (28), a professional antigen-presenting cell that promotes the initiation of an adaptive immune response. Thus, it is conceivable that as a consequence of decreased NKp30 expression, the process of NK-dependent DC maturation would be impaired in PAI patients, hampering the development of an antigen-specific adaptive immune response, one consequence of which would be delayed clearance of invading pathogens.

One potential explanation for the downregulation of activatory receptors on NK cells from PAI patients is a change in epigenetic regulation. In a recent study, Bjanesoy *et al.* (29) identified multiple hypomethylated gene promoter regions in CD4^+^ T cells isolated from PAI patients. Given that *in vitro* treatment of NK cells with demethylating agents has been shown to result in decreased surface expression of NKG2D (30), it would be of interest for future studies to examine the DNA methylation profile of NK cells from PAI patients, focusing primarily upon the promoters of genes that encode NK cell activatory receptors.

Of note, the polymorphic MHC class I polypeptide-related sequence A (MICA) molecule is a ligand for NKG2D and one of its variants, MICA5.1 or ICA*008, has been shown to be strongly associated with the risk of developing autoimmune Addison’s disease (31). This would suggest that the low NKCC we observed in our PAI patient cohort may be more related to the cause than to the consequences of PAI. However, we also included patients with PAI due to bilateral adrenalectomy, i.e., patients with no underlying autoimmune disorder, and this patient group, albeit small in size, showed similar and significant decreases in NKCC (Fig. 3B) and also NKG2D expression.

Taken together, we have shown for the first time that NKCC is significantly decreased in patients with PAI, providing a potential causal link explaining increased morbidity and mortality in PAI patients as a consequence of respiratory infections. Neither cause of disease nor DHEA replacement did influence the significant reduction in NKCC in PAI patients, which leads us to propose that the lack of diurnal cortisol delivery and consequently altered peripheral CLOCK gene regulation of immune cells may be the underlying causative mechanism. It will be highly interesting to examine whether introduction of glucocorticoid replacement with a delivery mode mimicking the physiological diurnal profile including the early morning cortisol surge (32, 33) could reestablish normal NKCC in adrenal insufficiency and help to reduce morbidity and ultimately mortality.

## Declaration of interest

W A and I B are consultants to Diurnal Ltd. All other authors have nothing to disclose.

## Funding

This work was supported by the Medical Research Council UK (Program Grant 0900567, to W A), the Mayo Clinic Foundation for Education and Research (Mayo Scholarship, to I B) and the Wellcome Trust (Clinical Research Training Fellowship WT101671, to V C).
